# The impact of age on load-related dorsolateral prefrontal cortex activation

**DOI:** 10.3389/fnagi.2014.00009

**Published:** 2014-02-05

**Authors:** Max Toepper, Helge Gebhardt, Eva Bauer, Anke Haberkamp, Thomas Beblo, Bernd Gallhofer, Martin Driessen, Gebhard Sammer

**Affiliations:** ^1^Research Department, Evangelic Hospital Bielefeld (EvKB)Bielefeld, Germany; ^2^Cognitive Neuroscience at Centre for Psychiatry, Justus-Liebig-University GiessenGiessen, Germany; ^3^Bender Institute of Neuroimaging, Justus-Liebig-University GiessenGiessen, Germany; ^4^Clinical Psychology and Psychotherapy, Philipps-University MarburgMarburg, Germany

**Keywords:** aging, CRUNCH, functional magnetic resonance imaging, prefrontal cortex, dorsolateral prefrontal cortex, spatial working memory, executive functioning, Corsi

## Abstract

Healthy aging is accompanied by working memory-related functional cerebral changes. Depending on performance accuracy and the level of working memory demands, older adults show task-related patterns of either increased or decreased activation compared to younger adults. Controversies remain concerning the interpretation of these changes and whether they already manifest in earlier decades of life. To address these issues, functional magnetic resonance imaging (fMRI) was used to examine brain activation during spatial working memory retrieval in 45 healthy individuals between 20 and 68 years of age. Participants performed a modified version of the Corsi Block-Tapping test (CBT). The CBT requires the storage and subsequent reproduction of spatial target sequences and allows modulating working memory load by a variation of sequence length. Results revealed that activation intensity at the lowest CBT load level increased with increasing age and positively correlated with the number of errors. At higher CBT load levels, activation intensity decreased with increasing age together with a disproportional accuracy decline on the behavioral level. Moreover, results suggests that younger individuals showed higher activation intensity at high CBT load than at low CBT load switching to the opposite pattern at an age of about 40 years. Consistent with the assumptions of the Compensation-Related Utilization of Neural Circuits Hypothesis (CRUNCH), the present results reveal specific age-related alterations in left dorsolateral prefrontal cortex activation in response to increasing task load. Specifically, the results point toward increasing neural inefficiency with age at low task load and a progressive limitation of resources with age at higher task load. The present findings argue for an increasing functional cerebral dysfunction over a time span of 50 years that may partly be compensated on the behavioral level until a resource ceiling is approached.

## Introduction

Since our environment provides a huge complexity of spatial information, we constantly need to adapt our spatial memory system. Thereby, we have to encode, maintain, update, and recall spatial information, as well as preserve these processes from distraction. Efficient coordination of these processes depends on intact spatial working memory operations which find their neural substrate in a complex anterior-posterior network of particularly posterior parietal, premotor, and prefrontal brain regions (Cabeza and Nyberg, 2000; Hartley and Speer, 2000; Rottschy et al., 2012). In addition, there is evidence for hippocampal and cerebellar involvement in spatial processing. Hippocampus and cerebellum both support spatial navigation (Burgess et al., 2002; Stoodley, 2012). Moreover, the hippocampus plays a major role for object-location associations and positional memory (Kessels et al., 2001; Bird and Burgess, 2008) while cerebellar brain regions are particularly associated with sequential information processing (Leggio et al., 2008; Tedesco et al., 2011). Posterior parietal brain activation within the superior parietal lobe and the precuneus is also linked to various working memory-related sub processes as storage of information (Hartley and Speer, 2000), mental representation of space (Bueti and Walsh, 2009) or effort (Allen et al., 2007; Morgan et al., 2007). In addition, posterior parietal and premotor cortices are involved in different forms of attentional processing as focused spatial attention and the shift of spatial attention toward and between locations (Simon et al., 2002; Constantinidis, 2006; Osaka et al., 2007). By contrast, higher-level working memory control processes are rather attributed to the prefrontal cortex (Funahashi, 2001; Egner and Hirsch, 2005; Collette et al., 2006). Moreover, the prefrontal cortex can be segregated into different functional sub regions (Petrides, 1994, 1995; Owen et al., 1996; D'Esposito et al., 1999). Whereas ventrolateral parts are involved in the maintenance of information, dorsolateral prefrontal activation particularly reflects working memory top-down control during updating, manipulation, and inhibition (D'Esposito et al., 1999; Smith and Jonides, 1999; Collette et al., 2006). Moreover, the extent of prefrontal activation—especially dorsolateral prefrontal activation—varies as a function of working memory demands (Cappell et al., 2010; Rottschy et al., 2012; Bennett et al., 2013). Toepper et al. (2010a), for example, showed that increasing task demands by requested spatial inhibition led to increased activation within exclusively dorsolateral parts of the left prefrontal cortex. By contrast, other authors assume a hierarchical rostro-caudal distinction of control functions in the frontal lobe with rostral parts being associated with cognitive control and caudal parts being related to spatial maintenance (Courtney et al., 1998; Badre and D'Esposito, 2009; Nee et al., 2013). However, both approaches particularly highlight the role of anterior parts of the dorsolateral prefrontal cortex in top-down working memory control.

In the recent decade, studies have shown that working memory declines across the life span even in the absence of disease-related cerebral pathology (Park et al., 2002; De Beni and Palladino, 2004; Holtzer et al., 2009). One reason for this decline certainly are working memory-related functional cerebral changes particularly within prefrontal cortex (Cabeza et al., 2004; Nagel et al., 2009; Reuter-Lorenz and Park, 2010). These changes can be classified into quantitative and qualitative changes. Quantitative activation changes imply that older individuals show either increased (“overactivation”) or decreased (“underactivation”) activation compared to younger individuals. Prefrontal “underactivation” in older individuals was often associated with functional deficits, particularly when additional resources had to be utilized due to increasing task complexity (Nagel et al., 2009; Cappell et al., 2010; Bennett et al., 2013). By contrast, prefrontal “overactivation” related to decreased or equivalent performance accuracy was interpreted as neural inefficiency, dedifferentiation of cortical subregions or compensation (Park et al., 2004; Zarahn et al., 2007; Carp et al., 2010). Qualitative activation changes are characterized by increased activation of basically task-irrelevant brain structures in older individuals. The most popular model concerning such qualitative changes is the Hemispheric Asymmetry Reduction in Old Adults (HAROLD) model (Cabeza, 2002). The HAROLD model postulates that older individuals show more bilateral activation compared to younger individuals. This bilateral recruitment was interpreted as dedifferentiation of cortical sub regions or as compensatory mechanism for age-related working memory decline (Cabeza, 2002; Park et al., 2004; Rajah and D'Esposito, 2005).

Taken together, many assumptions about the kind, direction and meaning of age-related functional cerebral changes were discussed in the last decade. Reuter-Lorenz and Cappell (2008) integrated these varying assumptions by presenting the Compensation-Related Utilization of Neural Circuits Hypothesis (CRUNCH). The CRUNCH model postulates that the kind of activation differences between older and younger individuals is strongly dependent on performance accuracy and on the cognitive demands of the applied task: At lower demand levels, older adults show comparable performances but more or bilateral (i.e., HAROLD) prefrontal activation compared to younger adults indicating compensatory mechanisms for age-related restraints in working memory capacity. At high task demands, these mechanisms fail resulting in poorer performances and decreased activation of prefrontal brain areas which reflects a working memory overload and restricted neural resources in older adults. Younger individuals, by contrast, show increased activation or bilaterality only at high task demands indicating a compensatory recruitment of neural resources similar to older adults at low task demands.

Another approach that integrates the varying results focuses on a dorsolateral-ventrolateral prefrontal cortex organization and region-specific changes with advancing age. Initially, it was proposed that age-related changes within dorsolateral and not ventrolateral parts of the prefrontal cortex are associated with the working memory decline during normal aging (Rypma and D'Esposito, 2000; Rypma et al., 2001). Rajah and D'Esposito (2005) specified these assumptions by attributing bilateral activation of ventrolateral prefrontal cortex to the dedifferentiation of cortical function, right dorsolateral prefrontal activation changes to functional deficits, and left dorsolateral prefrontal cortex activation changes to functional compensation.

Overall, there is a lot of evidence confirming load-related patterns of “overactivation” and “underactivation” in older individuals as proposed by the CRUNCH model. However, probably due to great heterogeneity of different theoretical and methodological approaches, there are heterogeneous assumptions about the meaning of these changes (e.g., neural inefficiency or compensation) and whether the CRUNCH assumptions are restricted to group differences between younger and older adults or may also apply to middle-aged individuals. To address these issues, functional magnetic resonance imaging (fMRI) was used to examine age-related brain activation changes during spatial working memory retrieval. Presuming the validity of the CRUNCH model, prefrontal cortex activation at low task demands should increase with advancing age (Reuter-Lorenz and Cappell, 2008; Reuter-Lorenz and Park, 2010), whereas prefrontal activation at high task demands should decrease with advancing age (Nagel et al., 2009; Cappell et al., 2010; Bennett et al., 2013). Moreover, we expect that younger individuals show higher activation intensity at high task demands than at low task demands whereas older individuals show the opposite pattern. Since particularly dorsolateral prefrontal cortex is recruited in response to increased working memory demands (Nagel et al., 2009; Toepper et al., 2010a) and since load-related dorsolateral prefrontal activation is affected by aging (Mattay et al., 2006; Cappell et al., 2010; Bennett et al., 2013), the age-related activation changes described above should predominantly manifest in dorsolateral parts of the prefrontal cortex.

## Materials and methods

### Participants

The study included 45 right-handed individuals (20 female, 25 male) with normal or corrected-to-normal vision. Participants were between 21 and 68 years of age (Table 1). None of the participants had a documented diagnosis of neurological or psychiatric disease in the past. Moreover, global cognitive deficits that might have pointed toward dementia were excluded by the Mini Mental State Examination (MMSE, Folstein et al., 1975) in individuals older than 45 years of age. Participants were recruited by local advertising in newspapers and provided a written declaration of consent prior to study start. The study obtained ethical approval by the Institutional Review Board of the University of Giessen.

**Table 1:** **Sample characteristics**.

*N*	45
Mean age (*SD*)	41.8 (13.5)
Age range	21–68
Gender (f/m)	20/25
Education: MWT (*SD*)	30.8 (2.9)
Education range	18–37

N, Number of participants; SD. Standard deviation; f, Female; m, Male; MWT, Vocabulary test; mean age and age range are in years.

### Educational differences

Since today's general school system and former systems differ from each other and with them the average time of received school education in years, the multiple choice vocabulary test MWT (Lehrl et al., 1995) was applied to exclude possible age-related intellectual and educational differences. The MWT is a valid and short German questionnaire to estimate crystallized intelligence. Its total score is a predictor for the level of education.

### Task and experimental procedure

**Figure 1:**
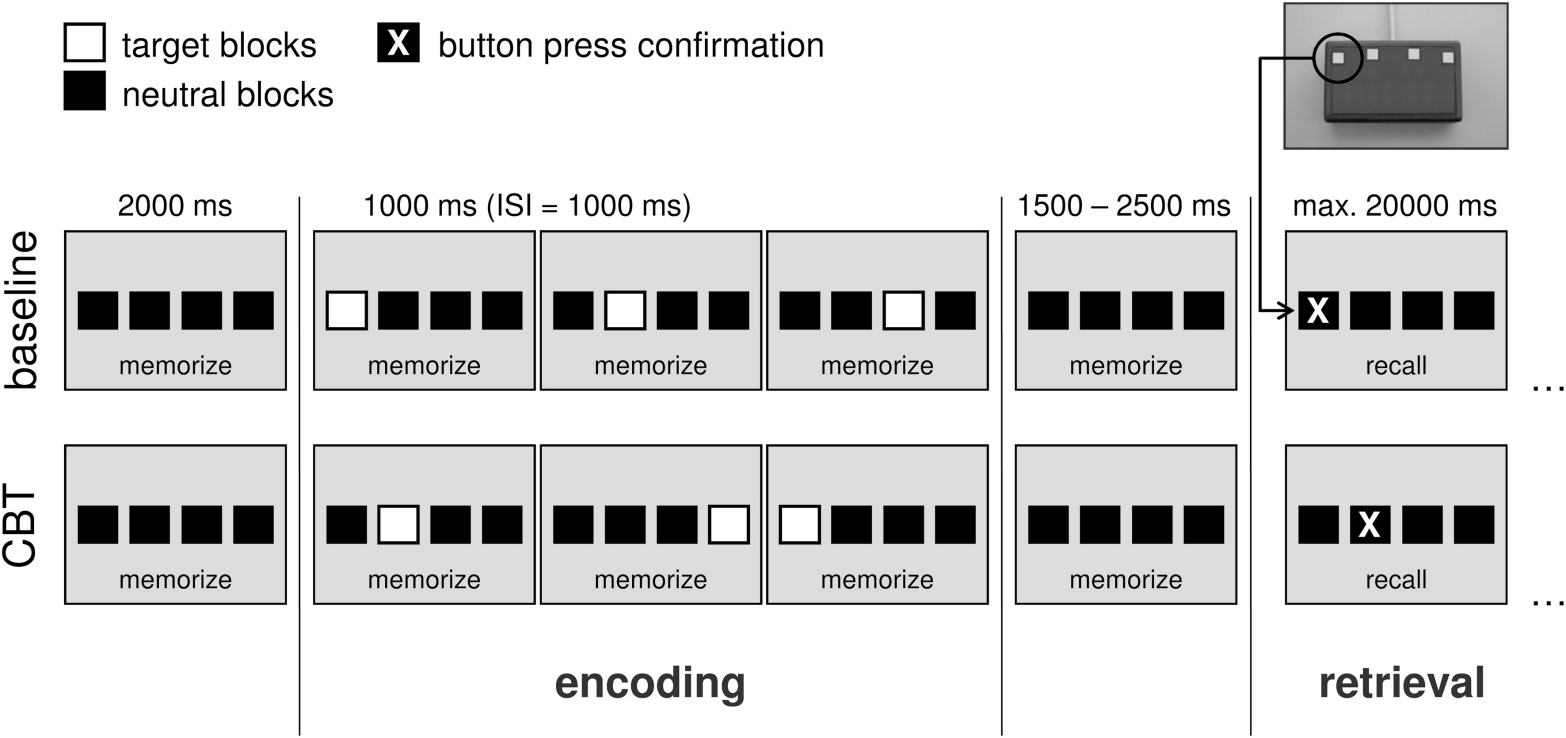
**Experimental design at an exemplary load level of 3.** In the baseline condition, target locations were presented from left to right. In the CBT condition, target blocks randomly changed their location. Duration of the target blocks was 1000 ms with a 1000 ms inter-stimulus interval. The encoding phase (stimulus presentation) was preceded by a pause of 2000 ms and followed by another pause of 1500–2500 ms (variable jitter). After that, participants were asked to reproduce the presented sequence by sequential button presses (retrieval phase). Available time for making responses was set to maximal 20,000 ms. After the final response, a fixcross was shown for the remaining time of the 20 s interval. (CBT, Corsi Block-Tapping test; ISI, inter-stimulus interval; ms, milliseconds; max., maximal).

To assess spatial working memory during fMRI, a modified electronical version of the Corsi Block-Tapping test (CBT; Corsi, 1972; Toepper et al., 2010a,b) was administered. The CBT is a multiple-item spatial working memory task that requires the storage and reproduction of spatial target sequences. Its original version provides nine different possible target locations on a board or a screen. Locations are randomly presented one after another and have to be reproduced in the correct temporal order immediately afterwards. Starting with two trials of three locations in a row, the difficulty level is continuously raised by one additional location until both trials of the same difficulty level are incorrect. The CBT is often used in clinical settings and well established with regard to psychometric properties. Moreover, this paradigm allows modulating working memory load by a variation of sequence length. The modified version provided four potential target locations (instead of nine as in the original version) indicated by four horizontally arranged blocks (Figure 1). Preliminary analyses revealed nearly identical whole-brain activation patterns at the original (Toepper et al., 2010a,b) and the modified (Toepper et al., 2013) block formation of the CBT in younger individuals indicating that the same cognitive and neural processes are involved (Supplementary Material_1).

Participants were asked to learn (encoding phase) and reproduce (retrieval phase) sequences of randomly presented target locations. Sequence length was varied between three (load 3), four (load 4), and five (load 5) locations in a row. In the baseline condition, all four target locations were presented from left to right. The chronological order of the different experimental conditions (baseline, load 3, load 4, load 5) was pseudorandomized. The sequence of presentation was equal for all participants. After the presentation of each sequence, participants were asked to reproduce this sequence by sequential button presses. Therefore, a keypad with four horizontally arranged buttons was designed. Each of these four buttons represented the corresponding block on the screen. As direct feedback for the participants (i.e., that they pressed one of the buttons), each button press was confirmed by a change of the respective block's color.

#### Stimulus material

In the modified version of the Corsi task, four horizontally arranged blocks were displayed on the screen. Stimulus material consisted of four black blocks (RGB 0 0 0) on gray background (RGB 163 163 163). Target blocks were displayed in natural-red (RGB 255 0 0). Response blocks were black (RGB 0 0 0) and turned to yellow (RGB 255 255 0) at button press.

#### Phases, durations, onsets

Each trial of the CBT can be subdivided into two main phases: an encoding phase (stimulus presentation) and a retrieval phase (probe). The encoding phase started with the onset of the first target block of every sequence and ended 1000 milliseconds (ms) after the end of the last one. Duration of the target blocks was 1000 ms with a 1000 ms inter-stimulus interval. Due to different load levels (3, 4, 5), the length of the encoding phase varied between 6000, 8000, and 10,000 ms. Each encoding phase was preceded by a pause of 2000 ms in which only the four horizontal blocks were shown. The time interval between the end of the encoding phase and the beginning of the retrieval phase was varied between 1500 and 2500 ms (variable jitter; Amaro and Barker, 2006). During this time interval, only the four horizontal blocks were shown. Available time for making responses (retrieval phase) was set to 20,000 ms. The length of this time period was determined based on the results of behavioral measures outside the scanner that indicated how long subjects would need at most to reproduce a sequence of a certain length. The retrieval phase lasted until the time of the final response. For the remaining time of the 20 s interval, a fixcross was shown.

Participants had to perform four trials per CBT sequence length as well as eight baseline trials. Consequently, 20 trials were randomly administered. In addition, participants completed five partial trials per condition (encoding phase only) in order to allow for the isolation of blood oxygenation level dependent (BOLD) signal-changes to the different phases of the task (Motes and Rypma, 2010). Total duration of the experiment was about 12 min.

#### Instructions and practice session

Before entering the MRI examination room, participants obtained precise instructions concerning the experimental procedure. Subjects were instructed to memorize the correct locations and temporal order of the presented target blocks. For retrieval, participants were advised to reproduce the presented target sequences by successive button presses and to respond as fast and as accurate as possible. In addition, subjects had to perform a series of practice trials on a PC outside the scanner. Practice trials included all load levels. Their chronological order was pseudorandomized and none of the trials was also used for the fMRI experiment. Duration of the practice session was 5 min.

### Data acquisition

Functional and structural images were acquired with a 1.5-Tesla Siemens Symphony whole-body scanner (Siemens, Erlangen, Germany) with a quantum gradient system and a standard 12-channel head coil (Siemens, Erlangen, Germany). Functional images were obtained using a single shot T2*-weighted gradient-echo planar imaging (EPI) sequence. The number of volumes was 210, each containing 30 transversal slices covering the whole brain and measured in descending order parallel to the AC-PC line (slice thickness = 4 mm; 1 mm gap; *TR* = 3000 ms; *TE* = 59 ms; flip angle = 90°; field of view = 192 × 192 mm^2^; matrix size = 64 × 64; voxel size = 3 × 3 × 4 mm^3^). During scanning, participants lay comfortably in a supine position in the MR scanner. An adjustable head holder restricted head movements. Visual stimuli were displayed on a back-projection screen (1280 × 1024 pixels) near the tube end. Subjects watched the screen via a dual-mirror which was mounted to the head coil. The angle of vision was approximately 11 degrees vertically and 18 degrees horizontally. Before the beginning of the experiment, high-resolution anatomical images were acquired using a T1-weighted, three-dimensional Magnetization Prepared Rapid Gradient Echo (MPRage) sequence.

### Data analysis

#### Behavioral data analysis

Effects of load and age on behavioral performance were analyzed using repeated measures analyses of variance (ANOVA) for the number of errors and mean retrieval durations per target. Within factor was “load” (load levels 3, 4, and 5), age was included as covariate. Possible effects of gender and education were controlled by including the between factor “gender” and a covariate representing the MWT total score. Since retrieval durations are usually not recorded in classic CBT, CBT retrieval durations have not frequently been analyzed so far. However, the fMRI-adapted mode for responding in this study allowed recording retrieval durations for each experimental condition. Retrieval durations were defined as the total amount of time needed to reproduce the sequences during the probe (time between probe start and last button press). Only correct trials (i.e., entire sequence correct) were included into the analysis of retrieval durations. Behavioral data were analyzed using SPSS Statistics 20.0. All levels of significance were α < 0.05 and two-tailed.

#### Brain data analysis

fMRI data were analyzed using SPM 8 (Statistical Parametric Mapping Software; Wellcome Institute of Neurology at University College London, UK. http://www.fil.ion.ucl.ac.uk/spm). The first three images of every EPI-recording session were discarded to account for the time needed for the magnetic field to achieve a steady state. The EPI-data were preprocessed including movement and slice time correction, 12 parameter non-linear normalization (2 × 2 × 2 mm) into the Montreal Neurological Institute (MNI) reference space, and smoothing (FWHM = 8 mm). Functional imaging data were analyzed using a general linear model (GLM) with four retrieval regressors (baseline retrieval, load 3 retrieval, load 4 retrieval, load 5 retrieval) and four encoding regressors (baseline encoding, load 3 encoding, load 4 encoding, load 5 encoding). To correct for errors, only correct trials were included into analysis. Timing of regressors followed the timing as explained in section “Phases, durations, onsets”. In addition, six movement regressors were included into the design. Regressors were convolved using the hemodynamic response function as provided in SPM 8. Design matrix was highpass filtered (128 s). Since the present study focused on age-related changes during spatial working memory retrieval, only the retrieval regressors were further analyzed. Retrieval-related activation at the different load levels was contrasted to retrieval baseline activation for each load level separately (contrast 1: load 3 > baseline, contrast 2: load 4 > baseline, contrast 3: load 5 > baseline). Moreover, the different retrieval regressors were modeled as linear trend from baseline to load 5 (contrast 4). All contrasts were first computed for each subject on the first level. Contrast 4 images were then applied to a second level random-effects group analysis (one sample *t*-test) to identify brain regions that are up-regulated in response to increasing task load across all participants. To examine age effects on load-related activation (load × age interaction), age was included as covariate of interest into the group analysis of contrast 4. Thereby, negative and positive age × load interaction effects were analyzed using contrast 4 with a −1 loading on the age covariate (contrast 5: negative load × age interaction), and with a +1 loading on the age covariate (contrast 6: positive load × age interaction) respectively.

To specify the negative age × load interaction within left dorsolateral prefrontal cortex, activation intensity was analyzed for the different load levels separately. Therefore, the coordinates (−38 36 28) of a brain region known to be associated with spatial working memory across different age groups and load levels (Nagel et al., 2009) were used to extract beta values from contrasts 1, 2, and 3 (see above) for each subject separately. These beta values represented BOLD signal changes from baseline to the three different load levels (in arbitrary units; Poldrack, 2007). Beta values for each subject and load level were then fed into a repeated measures ANOVA and analyzed using SPSS Statistics 20.0. Within factor was “load” (load levels 3, 4, and 5), age was included as covariate. As in the behavioral data analyses, the between factor “gender” and a covariate representing the MWT total score were included into the ANOVA to control for possible effects of gender and education on activation intensity.

***ROI analyses***. Brain activation was analyzed at whole brain level and by a region of interest (ROI) approach. ROI analyses comprised only a priori chosen brain regions. Particularly Brodmann area (BA) 9 and BA 46 within dorsolateral prefrontal cortex are recruited in response to increasing working memory demands (Mattay et al., 2006; Nagel et al., 2009; Cappell et al., 2010; Toepper et al., 2010a). Next to these brain regions, BAs 44 and 45 (ventrolateral prefrontal cortex), BA 6 (premotor cortex), and BA 7 (posterior parietal cortex) as prominent parts of the working memory network showed increased activation at higher demand levels (Nagel et al., 2009, 2011; Prakash et al., 2009). In addition, the up-regulation of particularly dorsolateral prefrontal cortex in response to increasing task load is affected by age (Mattay et al., 2006; Nagel et al., 2009; Bennett et al., 2013). Based on these findings and the theoretical considerations in the introduction section, the mentioned regions were included into ROI analyses. Data were analyzed using the corresponding ROI masks of the automated anatomical labeling atlas (AAL; Tzourio-Mazoyer et al., 2002) which is implemented in the WFU PickAtlas (Maldjian et al., 2003), an automated software toolbox for generating ROI masks based on the Talairach Daemon database (Talairach and Tournoux, 1988; Lancaster et al., 1997, 2000). All reported ROI results were tested at a local significance threshold of *p* < 0.05 (voxel level). Alpha adjustment for multiple comparisons was done for each ROI (family-wise error (FWE) correction). Bonferroni adjustments for the number of tested ROIs are optionally provided in the results section. Whole-brain results were tested using a threshold of *Z* > 3.1 with a minimum cluster size of 20 voxels and a cluster significance threshold of *p* < 0.05 (FWE-corrected for multiple comparisons).

***Activation intensity × performance accuracy***. To get information about the relation between BOLD signal changes and performance accuracy at different load levels, contrast values of the peak voxel identified by the analysis described above (see section “Brain data analysis”) were correlated with the number of errors for each load level separately. Since age is associated with both, higher activation intensity and lower performance accuracy, the correlation between activation intensity and performance accuracy was controlled for age (partial correlation, Pearson).

## Results

### Behavioral data

None of the participants older than 45 years of age showed an MMSE score lower than 27 points (mean: 28.7 ± 1.0; range: 27–30), severe cognitive deficits that might have pointed toward dementia could therefore be ruled out.

#### Errors

**Figure 2:**
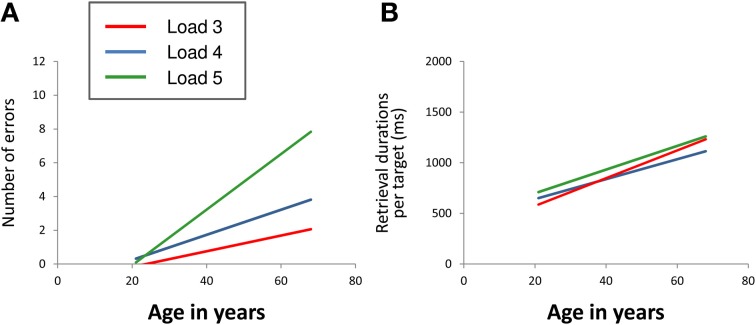
Linear relation between age and mean number of errors **(A)** and between age and mean retrieval durations per target **(B)** at CBT load levels 3 (red line), 4 (blue line), and 5 (green line). The regression lines show that the number of errors increased with increasing age. At load level 4, there was a sharper increase than at load level 3 with the sharpest increase at load level 5 (load × age interaction). Mean retrieval durations per target also increased with increasing age. The extent of increase did not differ between the different load levels (CBT, Corsi Block-Tapping test; ms, milliseconds).

For the number of errors (Figure 2A), repeated measures ANOVA revealed a significant main effect of “age” [*F*_(1, 39)_ = 19.66, *p* < 0.001] as well as a significant “load” × “age” interaction effect [*F*_(2, 78)_ = 10.96, *p* < 0.001]. Main effects of “load” [*F*_(2, 78)_ = 0.72, *p* = 0.488], “gender” [*F*_(1, 39)_ = 0.06, *p* = 0.812] and “education” [*F*_(1, 39)_ = 3.03, *p* = 0.090] as well as the interaction effects “load” × “gender” [*F*_(2, 78)_ = 0.02, *p* = 0.982] and “load” × “education” [*F*_(2, 78)_ = 2.17, *p* = 0.121] did not reach significance. The results revealed that the number of errors increased with increasing age. At load level 4, there was a sharper increase than at load level 3 with the sharpest increase at load level 5. Gender and education effects on performance accuracy were not found.

#### Mean retrieval durations per target

For mean retrieval durations per target (Figure 2B), repeated measures ANOVA revealed a significant main effect of “age” [*F*_(1, 39)_ = 36.31, *p* < 0.001]. Main effects of “load” [*F*_(2, 78)_ = 1.28, *p* = 0.283], “gender” [*F*_(1, 39)_ = 1.83, *p* = 0.184] and “education” [*F*_(1, 39)_ = 1.29, *p* = 0.263] as well as the interaction effects “load” × “age” [*F*_(2, 78)_ = 1.51, *p* = 0.227], “load” × “gender” [*F*_(2, 78)_ = 2.18, *p* = 0.120] and “load” × “education” [*F*_(2, 78)_ = 0.78, *p* = 0.461] did not reach statistical significance at *p* < 0.05. The results revealed increasing retrieval durations per target with increasing age. The extent of increase did not differ between the different load levels. Gender and education effects on retrieval durations per target were not found either.

### fMRI data

#### The impact of age on load-related brain activation

**Figure 3:**
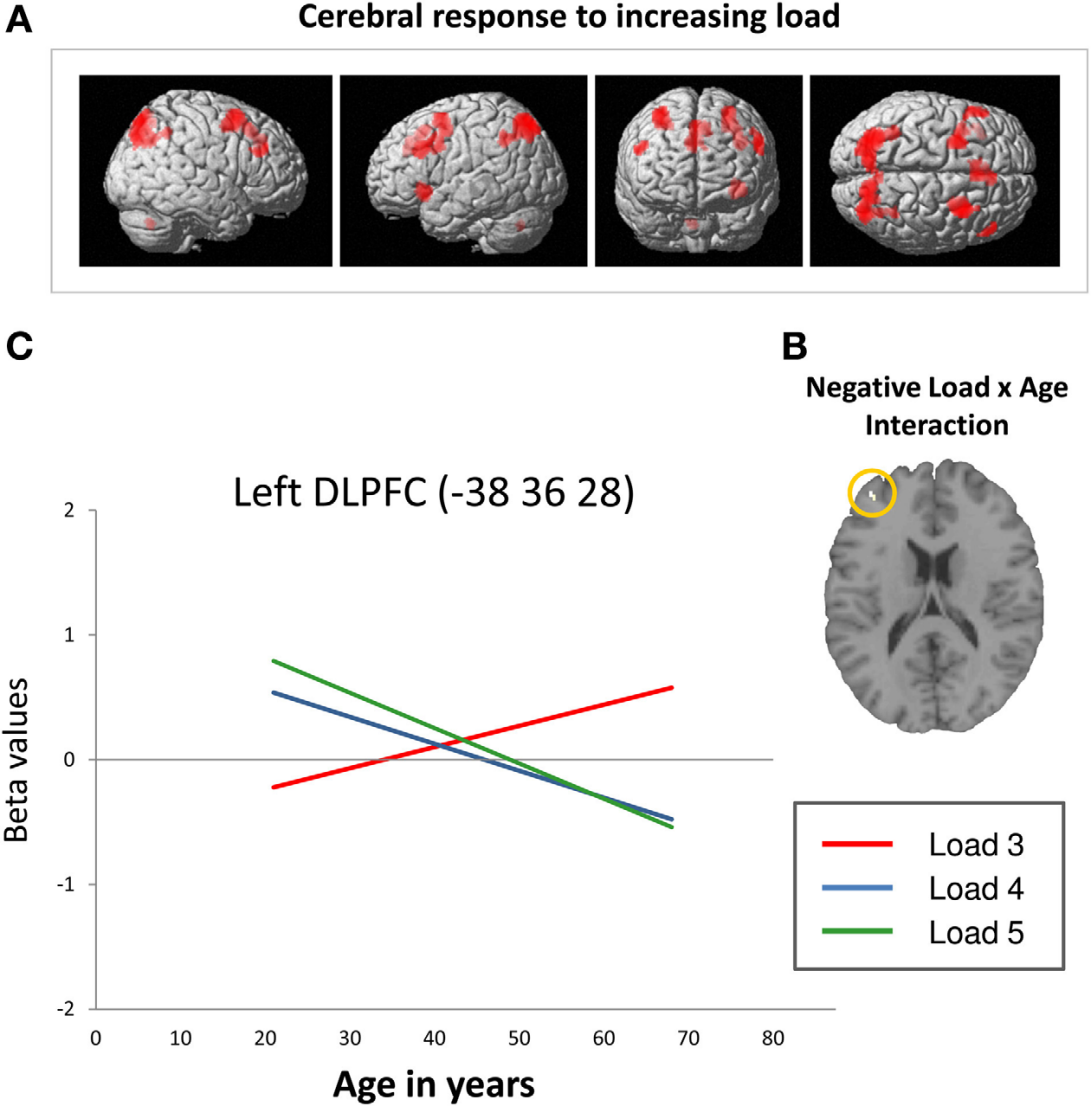
**(A)** Increasing frontal, parietal and cerebellar activation in response to increasing task load across all participants at a cluster significance threshold of *p* < 0.05 (FWE-corrected for multiple comparisons). **(B)** Decreasing retrieval-related DLPFC activation with increasing age in response to increasing task load (negative load × age interaction; *p* < 0.05, FWE-corrected). **(C)** Linear relation between retrieval-related BOLD signal change (in arbitrary units; Poldrack, 2007) within the left DLPFC (−38 36 28; Nagel et al., 2009) and age at CBT load levels 3 (red line), 4 (blue line), and 5 (green line). The regression lines show that activation intensity increased with increasing age at load level 3 and decreased with increasing age at load levels 4 and 5 (DLPFC, dorsolateral prefrontal cortex; BA, Brodmann area).

Across all participants, data analysis revealed an up-regulation of frontal, parietal, and cerebellar brain regions in response to increasing task demands (Figure 3A). Specifically, ROI analyses showed increased activation intensity within left dorsolateral prefrontal cortex, bilateral premotor cortex, and bilateral posterior parietal cortex (Table 2). Whereas a positive load × age interaction is not reflected by the present data, brain data analysis revealed a significant negative load × age interaction (contrast 5): ROI analyses identified a cluster within anterior BA 9 of the left dorsolateral prefrontal cortex whose activation intensity increased with decreasing age (Figure 3B, Table 3). Repeated measures ANOVA for signal change within left dorsolateral prefrontal cortex (−38 36 28) also revealed a significant “load” × “age” interaction effect [*F*_(2, 78)_ = 7.09, *p* = 0.001]. Main factors “age” [*F*_(1, 39)_ = 1.80, *p* = 0.187], “load” [*F*_(2, 78)_ = 0.15, *p* = 0.864], “gender” [*F*_(1, 39)_ = 0.01, *p* = 0.936] and “education” [*F*_(1, 39)_ = 1.23, *p* = 0.275] as well as the interaction effects “load” × “gender” [*F*_(2, 78)_ = 0.41, *p* = 0.669] and “load” × “education” [*F*_(2, 78)_ = 1.55, *p* = 0.220] did not reach statistical significance at *p* < 0.05.

**Table 2:** **Cerebral activation associated with increasing task load during CBT retrieval across all participants**.

**ROI**	**Hemisphere**	**Anatomical region**	**Number of activated voxels**	**Peak MNI coordinates**			***t*-value**	**p-value (FWE-corrected)**	***p*-value (conservative Bonferroni-correction for set of ROIs)**
				***x***	***y***	***z***			
BA 9	L	Middle frontal gyrus	28	−52	26	34	4.67	0.008	0.096
BA 6	L	Middle frontal gyrus	81	−26	2	54	5.79	0.001	0.012
BA 6	R	Middle frontal gyrus	102	32	10	58	5.74	0.001	0.012
BA 7	L	Superior parietal lobule	105	−28	−72	52	5.70	<0.001	<0.010
BA 7	R	Superior parietal lobule	58	20	−74	54	5.26	0.002	0.024

CBT, Corsi Block-Tapping test; ROI, Region of interest; BA, Brodmann area; MNI, Montreal Neurological Institute; FWE, Family-wise error; L, Left hemisphere; R, Right hemisphere.

**Table 3:** **Cerebral activation associated with a negative load × age interaction during CBT retrieval**.

**ROI**	**Hemisphere**	**Anatomical region**	**Number of activated voxels**	**Peak MNI coordinates**			***t*-value**	***p*-value (FWE-corrected)**	***p*-value (conservative Bonferroni-correction for set of ROIs)**
				***x***	***y***	***z***			
BA 9	L	Middle frontal gyrus	14	−40	50	18	4.64	0.023	0.092

CBT, Corsi Block-Tapping test; ROI, region of interest; BA, Brodmann area; MNI, Montreal Neurological Institute; FWE, family-wise error; L, left hemisphere.

Summarizing, the results revealed a positive slope of the linear regression of increasing activation intensity with increasing age at load level 3 and a negative slope of decreasing activation intensity with increasing age at load levels 4 and 5 (Figure 3C, please also see Supplementary Material_2). Moreover, Figure 3C suggests that younger individuals show higher activation intensity at load levels 4 and 5 than at load level 3 switching to the opposite pattern at an age of about 40 years.

***Activation intensity × performance accuracy***. After being controlled for age, left BA 9 signal change at CBT load level 3 positively correlated with the number of CBT errors at load level 3 (*r*: 0.332; *p* = 0.026). At the other load levels (load 4, load 5), there were no significant correlations with the number of errors in the respective condition.

## Discussion

In the present study, fMRI was used to investigate the impact of task load and age on spatial working memory performance and retrieval-related brain activation. Results are in line with the assumptions of the CRUNCH model about load-dependent patterns of prefrontal activation changes in older individuals. Activation intensity increased with increasing age at low task demands and decreased with increasing age at high task demands. As expected, the present findings additionally suggest that this load-related dissociation in activation intensity is not restricted to comparisons between younger and older individuals but, to a lesser degree, already be detectable in other decades of life. Moreover, aging seems to affect particularly rostral parts of the left dorsolateral prefrontal cortex with higher activity at low task load probably reflecting neural inefficiency or failed compensation.

### Spatial working memory performance

Analysis of behavioral data revealed that retrieval durations per target increased with increasing age. This finding confirms a progressive slowdown with advancing age which can be due to decreasing motor skills and mental processing speed (Park et al., 2002; Kumar and Foster, 2007; Seidler et al., 2010). However, the analysis of retrieval durations per target did not reveal a significant age × load interaction indicating that the increase of retrieval durations with age was equal at each load level. Consequently, first level contrasts should have been free of slowing-associated brain activation. The number of errors also increased with increasing age. Moreover, increasing load provoked a disproportional decline of working memory performance accuracy with advancing age. At load level 4, there was a more distinct accuracy decline with age than at load level 3 with the sharpest decline at load level 5. Together, these findings suggest a specific age-related spatial working memory dysfunction at increasing task load which supports previous studies that reported accretive working memory deficits across the life span in individuals without cerebral pathology, particularly when additional cognitive resources had to be utilized in response to increasing task demands (Nagel et al., 2009; Cappell et al., 2010; Bennett et al., 2013).

### Prefrontal brain activation and the impact of task demands

Also consistent with previous findings (Nagel et al., 2009; Rottschy et al., 2012), increasing task load provoked an up-regulation of prefrontal, parietal and cerebellar parts of the spatial working memory network across all participants (Figure 3A). Moreover, the up-regulation of these brain regions was affected by age. Brain data analysis identified a cluster within anterior BA 9 of the left dorsolateral prefrontal cortex in which load-associated activation intensity during working memory retrieval decreased with increasing age (Figure 3B). Analysis of local BOLD signal changes using an independent ROI within left dorsolateral prefrontal cortex (spatial working memory related brain region identified by Nagel et al., 2009) indicated how the direction of age-related activation differences varied depending on the level of working memory load: At higher load levels (load levels 4 and 5), activation intensity decreased with increasing age. Together with decreasing performance accuracy on the behavioral level, this finding probably indicates an age-related reduction of working memory resources at high task demands. At the lowest load level (load level 3), by contrast, activation intensity increased with increasing age (Figure 3C). Moreover, results revealed a positive correlation between activation intensity at load level 3 and the number of errors at load level 3 indicating that dorsolateral prefrontal activation intensity at low task demands rather reflects inefficient neural processing or a counterproductive try to recruit working memory resources than successful compensation. Moreover, Figures 2A and 3C suggest that younger individuals showed higher activation intensity at load levels 4 and 5 than at load level 3 but hardly any accuracy differences between these load levels. Consequently, these findings point toward a successful and flexible control of working memory resources in response to increasing task load in younger individuals (Nagel et al., 2009, 2011). Older individuals, by contrast, showed lower activation intensity at load levels 4 and 5 than at load level 3 as well as lower performance accuracy. Moreover, the extent of these differences increased with increasing age suggesting an advancing age-related reduction of working memory resources. When task demands get too complex, this dysfunction may lead to a working memory overload and a performance breakdown on the behavioral level (Reuter-Lorenz and Park, 2010; Nagel et al., 2011; Prakash et al., 2012).

The described dissociation illustrates the impact of task load on age-related activation differences and explains why brain data analysis did not reveal a significant main effect of age. In fact, age effects on activation intensity seem to average out across the different load levels (increasing activation with age at low task load and decreasing activation with age at high task load). Consequently, dorsolateral prefrontal brain activation with age does not seem to be affected by aging in general but is strongly modulated by the level of working memory demands (Prakash et al., 2009; Reuter-Lorenz and Park, 2010). Noteworthy, there was no main effect of load either because younger individuals showed higher activation intensity at high task load than at low task load, whereas older individuals showed the opposite pattern.

With the restriction of a limited generalizability of cross-sectional results, the present data finally point toward an increasing functional cerebral dysfunction over a time span of 50 years that may partly be compensated on the behavioral level until a resource ceiling is approached.

### Prefrontal brain activation and theories of aging

Our results mostly confirm the assumptions of the CRUNCH model (Reuter-Lorenz and Cappell, 2008; Reuter-Lorenz and Park, 2010; Schneider-Garces et al., 2010). As mentioned above, this model posits that older individuals compared to younger individuals show reduced prefrontal activation and lower performance accuracy at high task difficulty indicating limited working memory resources. At low task demands, by contrast, older individuals show equivalent performances as younger individuals but higher activation intensity as compensatory neural mechanism for age-related restraints in working memory capacity. The present findings are in line with the CRUNCH assumptions concerning this dissociation. In fact, activation intensity at low task load increased with advancing age whereas activation intensity at high task load decreased. At low task demands, however, the present results revealed a negative correlation between activation intensity and performance accuracy and therefore rather point toward inefficient neural processing or failed compensation than toward a successful compensatory recruitment of working memory resources. In addition, our data suggests that the CRUNCH assumptions about load-related discrepancies in activation intensity may not be restricted to differences between younger and older individuals but, to a lesser degree, also apply to other decades of life.

### Prefrontal cortex organization

Moreover, the present findings support the assumption of region-specific changes in prefrontal cortex organization with age. As mentioned above, the frontal lobe can be divided into different hierarchical functional modules with particularly rostral parts of the dorsolateral prefrontal cortex being associated with working memory control, whereas more caudal or ventrolateral prefrontal cortex activation is rather attributed to spatial maintenance (D'Esposito et al., 1999; Badre and D'Esposito, 2009; Nee et al., 2013). Moreover, it was stated that particularly dorsolateral and not ventrolateral prefrontal cortex is affected by age (Rypma and D'Esposito, 2000; Rypma et al., 2001). The present results confirm a distinct vulnerability of rostral parts of the dorsolateral prefrontal cortex in older individuals, whereas more caudal and ventrolateral parts seem to be preserved from age-related changes. Consequently, the present findings suggest a growing working memory control dysfunction with advancing age, whereas the capability to keep spatial information online may stay unimpaired. As outlined in the introduction section, Rajah and D'Esposito (2005) specified their assumptions concerning age-related dorsolateral prefrontal activation changes by attributing right-hemispheric alterations to functional deficits and left-hemispheric changes to functional compensation. In line with previous findings (Mattay et al., 2006; Nagel et al., 2009, 2011), the present results rather suggest an association between left dorsolateral prefrontal “underactivation” and functional deficits at high working memory demands and between left dorsolateral prefrontal “overactivation” and neural inefficiency or failed compensation at low working memory demands.

### Other brain regions associated with spatial working memory

Interestingly, age-related activation changes at increasing task load were only found within left dorsolateral prefrontal cortex whereas the load-dependent up-regulation of posterior parts of the spatial working memory network (e.g., posterior parietal cortex, premotor cortex) was not affected by age. This finding suggests that the working memory dysfunction in older individuals is restricted to the flexible control of working memory resources at increasing task complexity. The present results confirm that the recruitment of these resources relies on the functional integrity of the left dorsolateral prefrontal cortex which seems to be more and more limited with advancing age (Nagel et al., 2009; Prakash et al., 2012).

## Conclusions

In summary, the present results reveal brain activation changes with age during working memory retrieval. These changes particularly affect rostral parts of the left dorsolateral prefrontal cortex. In line with the assumptions of the CRUNCH model, the functional dorsolateral prefrontal alterations are modulated by the level of working memory demands and only occur when additional working memory resources have to be recruited in response to increasing task load.

At a low demand level, left-hemispheric dorsolateral prefrontal activation intensity increased with increasing age probably reflecting neural inefficiency or failed compensation. At higher task load, activation intensity decreased with increasing age together with a disproportional performance decline on the behavioral level. Latter findings suggest a limitation of working memory resources in older individuals at high task demands whereas younger individuals seem to successfully recruit additional resources in response to increasing task load.

In contrast to the CRUNCH assumptions, successful compensatory processes at low task demands are not reflected by the data. One reason for this discrepancy might be that the present results only refer to working memory retrieval. Definite conclusions about working memory encoding or maintenance processes cannot be drawn since serial processing paradigms as the CBT simultaneously require encoding and maintenance. Concurrently, encoding and maintenance are confounded which would have made an interpretation of activation differences complicated and made us focusing on the retrieval phase of the CBT. However, on the behavioral level it seems plausible that compensatory processes particularly manifest during encoding and maintenance (e.g., strategy, rehearsal) whereas the retrieval phase may rather indicate whether compensatory processes during encoding and maintenance were successful. Finally, longitudinal analyses are necessary to draw definite conclusions about activation changes with age. All of these considerations should be taken into account in future research.

### Conflict of interest statement

The authors declare that the research was conducted in the absence of any commercial or financial relationships that could be construed as a potential conflict of interest.

## References

[B1] Allen M. D. Bigler E. D. Larsen J. Goodrich-Hunsaker N. J. Hopkins R. O. (2007). Functional neuroimaging evidence for high cognitive effort on the Word Memory Test in the absence of external incentives. Brain Inj. 21, 1425–1428 10.1080/02699050701769819 18066945

[B2] Amaro E. Jr. Barker G. J. (2006). Study design in fMRI: basic principles. Brain Cogn. 60, 220–232 10.1016/j.bandc.2005.11.009 16427175

[B3] Badre D. D'Esposito M. (2009). Is the rostro-caudal axis of the frontal lobe hierarchical? Nat. Rev. Neurosci. 10, 659–669 10.1038/nrn2667 19672274 PMC3258028

[B4] Bennett I. J. Rivera H. G. Rypma B. (2013). Isolating age-group differences in working memory load-related neural activity: assessing the contribution of working memory capacity using a partial-trial fMRI method. Neuroimage 72, 20–32 10.1016/j.neuroimage.2013.01.030 23357076 PMC3602125

[B5] Bird C. M. Burgess N. (2008). The hippocampus and memory: insights from spatial processing. Nat. Rev. Neurosci. 9, 182–194 10.1038/nrn2335 18270514

[B6] Bueti D. Walsh V. (2009). The parietal cortex and the representation of time, space, number and other magnitudes. Philos. Trans. R. Soc. Lond. Biol. 364, 1831–1840 10.1098/rstb.2009.0028 19487186 PMC2685826

[B7] Burgess N. Maguire E. A. O'Keefe J. (2002). The human hippocampus and spatial and episodic memory. Neuron 35, 625–641 10.1016/S0896-6273(02)00830-9 12194864

[B8] Cabeza R. (2002). Hemispheric asymmetry reduction in older adults: the HAROLD model. Psychol. Aging. 17, 85–100 10.1037/0882-7974.17.1.85 11931290

[B9] Cabeza R. Daselaar S. M. Dolcos F. Prince S. E. Budde M. Nyberg L. (2004). Task-independent and task-specific age effects on brain activity during working memory, visual attention and episodic retrieval. Cereb. Cortex 14, 364–375 10.1093/cercor/bhg133 15028641

[B10] Cabeza R. Nyberg L. (2000). Imaging cognition II: an empirical review of 275 PET and fMRI studies. J. Cogn. Neurosci. 12, 1–47 10.1162/08989290051137585 10769304

[B11] Cappell K. A. Gmeindl L. Reuter-Lorenz P. A. (2010). Age differences in prefrontal recruitment during verbal working memory maintenance depend on memory load. Cortex 46, 462–473 10.1016/j.cortex.2009.11.009 20097332 PMC2853232

[B12] Carp J. Gmeindl L. Reuter-Lorenz P. A. (2010). Age differences in the neural representation of working memory revealed by multi-voxel pattern analysis. Front. Hum. Neurosci. 4:217 10.3389/fnhum.2010.00217 21151373 PMC2996172

[B13] Collette F. Hogge M. Salmon E. Van der Linden M. (2006). Exploration of the neural substrates of executive functioning by functional neuroimaging. Neuroscience 139, 209–221 10.1016/j.neuroscience.2005.05.035 16324796

[B14] Constantinidis C. (2006). Posterior parietal mechanisms of visual attention. Rev. Neurosci. 17, 415–427 10.1515/revneuro.2006.17.4.415 17139842

[B15] Corsi P. M. (1972). Human memory and the medial temporal region of the brain. Diss. Abstr. Int. 34, 819B

[B16] Courtney S. M. Petit L. Maisog J. M. Ungerleider L. G. Haxby J. V. (1998). An area specialized for spatial working memory in human frontal cortex. Science 279, 1347–1351 10.1126/science.279.5355.1347 9478894

[B17] De Beni R. Palladino P. (2004). Decline in working memory updating through ageing: intrusion error analyses. Memory 12, 75–89 10.1080/09658210244000568 15098622

[B18] D'Esposito M. Postle B. R. Ballard D. Lease J. (1999). Maintenance versus manipulation of information held in working memory: an event-related fMRI study. Brain Cogn. 41, 66–86 10.1006/brcg.1999.1096 10536086

[B19] Egner T. Hirsch J. (2005). The neural correlates and functional integration of cognitive control in a Stroop task. Neuroimage 24, 539–547 10.1016/j.neuroimage.2004.09.007 15627596

[B20] Finke K. Bublak P. Zihl J. (2006). Visual spatial and visual pattern working memory: neuropsychological evidence for a differential role of left and right dorsal visual brain. Neuropsychologia 44, 649–661 10.1016/j.neuropsychologia.2005.06.015 16111725

[B21] Folstein M. F. Folstein S. E. McHugh P. R. (1975). ‘Mini-mental state.’ A practical method for grading the cognitive state of patients for the clinician. J. Psychiatr. Res. 12, 189–198 10.1016/0022-3956(75)90026-6 1202204

[B22] Funahashi S. (2001). Neuronal mechanisms of executive control by the prefrontal cortex. Neurosci. Res. 39, 147–165 10.1016/S0168-0102(00)00224-8 11223461

[B23] Hartley A. A. Speer N. K. (2000). Locating and fractionating working memory using functional neuroimaging: storage, maintenance, and executive functions. Microsc. Res. Tech. 51, 45–53 10.1002/1097-0029(20001001)51:1<45::AID-JEMT5>3.0.CO;2-O 11002352

[B24] Holtzer R. Rakitin B. C. Steffener J. Flynn J. Kumar A. Stern Y. (2009). Age effects on load-dependent brain activations in working memory for novel material. Brain Res. 1249, 148–161 10.1016/j.brainres.2008.10.009 18983833 PMC2677982

[B25] Kessels R. P. d'Alfonso A. A. Postma A. de Haan E. H. (2000). Spatial working memory performance after high-frequency repetitive transcranial magnetic stimulation of the left and right posterior parietal cortex in humans. Neurosci. Lett. 287, 68–70 10.1016/S0304-3940(00)01146-0 10841993

[B26] Kessels R. P. de Haan E. H. Kappelle L. J. Postma A. (2001). Varieties of human spatial memory: a meta-analysis on the effects of hippocampal lesions. Brain Res. Brain Res. Rev. 35, 295–303 10.1016/S0165-0173(01)00058-3 11423159

[B27] Kumar A. Foster T. C. (2007). Neurophysiology of old neurons and synapses, in Brain Aging: Models, Methods, and Mechanisms, ed Riddle D. R. (Boca Raton, FL: CRC Press), 229–250 21204354

[B28] Lancaster J. L. Rainey L. H. Summerlin J. L. Freitas C. S. Fox P. T. Evans A. C. (1997). Automated labeling of the human brain: a preliminary report on the development and evaluation of a forward-transform method. Hum. Brain. Mapp. 5, 238–242 10.1002/(SICI)1097-0193(1997)5:4<238::AID-HBM6>3.0.CO;2-4 20408222 PMC2860189

[B29] Lancaster J. L. Woldorff M. G. Parsons L. M. Liotti M. Freitas C. S. Rainey L. (2000). Automated Talairach atlas labels for functional brain mapping. Hum. Brain. Mapp. 10, 120–131 10.1002/1097-0193(200007)10:3<120::AID-HBM30>3.0.CO;2-8 10912591 PMC6871915

[B30] Leggio M. G. Tedesco A. M. Chiricozzi F. R. Clausi S. Orsini A. Molinari M. (2008). Cognitive sequencing impairment in patients with focal or atrophic cerebellar damage. Brain 131, 1332–1343 10.1093/brain/awn040 18334535

[B31] Lehrl S. Triebig G. Fischer B. (1995). Multiple choice vocabulary test MWT as a valid and short test to estimate premorbid intelligence. Acta Neurol. Scand. 91, 335–345 10.1111/j.1600-0404.1995.tb07018.x 7639062

[B32] Maldjian J. A. Laurienti P. J. Kraft R. A. Burdette J. H. (2003). An automated method for neuroanatomic and cytoarchitectonic atlas-based interrogation of fMRI data sets. Neuroimage 19, 1233–1239 10.1016/S1053-8119(03)00169-1 12880848

[B33] Mattay V. S. Fera F. Tessitore A. Hariri A. R. Berman K. F. Das S. (2006). Neurophysiological correlates of age-related changes in working memory capacity. Neurosci. Lett. 392, 32–37 10.1016/j.neulet.2005.09.025 16213083

[B34] Morgan R. M. Parry A. M. Arida R. M. Matthews P. M. Davies B. Castell L. M. (2007). Effects of elevated plasma tryptophan on brain activation associated with the Stroop task. Psychopharmacology 190, 383–389 10.1007/s00213-006-0609-7 17180619

[B35] Motes M. A. Rypma B. (2010). Working memory component processes: isolating BOLD signal changes. Neuroimage 49, 1933–1941 10.1016/j.neuroimage.2009.08.054 19732840 PMC2804888

[B36] Nagel I. E. Preuschhof C. Li S. C. Nyberg L. Bäckman L. Lindenberger U. (2009). Performance level modulates adult age differences in brain activation during spatial working memory. Proc. Natl. Acad. Sci. U.S.A. 106, 22552–22557 10.1073/pnas.0908238106 20018709 PMC2799744

[B37] Nagel I. E. Preuschhof C. Li S. C. Nyberg L. Bäckman L. Lindenberger U. (2011). Load modulation of BOLD response and connectivity predicts working memory performance in younger and older adults. J. Cogn. Neurosci. 23, 2030–2045 10.1162/jocn.2010.21560 20828302

[B38] Nee D. E. Brown J. W. Askren M. K. Berman M. G. Demiralp E. Krawitz A. (2013). A meta-analysis of executive components of working memory. Cereb. Cortex 23, 264–282 10.1093/cercor/bhs007 22314046 PMC3584956

[B39] Nyberg L. Dahlin E. Stigsdotter Neely A. Bäckman L. (2009). Neural correlates of variable working memory load across adult age and skill: dissociative patterns within the fronto-parietal network. Scand. J. Psychol. 50, 41–46 10.1111/j.1467-9450.2008.00678.x 18705668

[B40] Osaka M. Komori M. Morishita M. Osaka N. (2007). Neural bases of focusing attention in working memory: an fMRI study based on group differences. Cogn. Affect. Behav. Neurosci. 7, 130–139 10.3758/CABN.7.2.130 17672384

[B41] Owen A. M. Evans A. C. Petrides M. (1996). Evidence for a two-stage model of spatial working memory processing within the lateral frontal cortex: a positron emission tomography study. Cereb. Cortex 6, 31–38 10.1093/cercor/6.1.31 8670636

[B42] Park D. C. Lautenschlager G. Hedden T. Davidson N. S. Smith A. D. Smith P. K. (2002). Models of visuospatial and verbal memory across the adult life span. Psychol. Aging 17, 299–320 10.1037/0882-7974.17.2.299 12061414

[B43] Park D. C. Polk T. A. Park R. Minear M. Savage A. Smith M. R. (2004). Aging reduces neural specialization in ventral visual cortex. Proc. Natl. Acad. Sci. U.S.A. 101, 13091-13095 10.1073/pnas.0405148101 15322270 PMC516469

[B44] Petrides M. (1994). Frontal lobes and working memory: evidence from investigations of the effects of cortical excisions in nonhuman primates, in Handbook of Neuropsychology, eds Boller F. Grafman J. (Amsterdam: Elsevier), 59–82

[B45] Petrides M. (1995). Functional organization of the human frontal cortex for mnemonic processing. Evidence from neuroimaging studies. Ann. N.Y. Acad. Sci. 769, 85–96 10.1111/j.1749-6632.1995.tb38133.x 8595046

[B46] Poldrack R. A. (2007). Region of interest analysis for fMRI. Soc. Cogn. Affect. Neurosci. 2, 67–70 10.1093/scan/nsm006 18985121 PMC2555436

[B47] Prakash R. S. Erickson K. I. Colcombe S. J. Kim J. S. Voss M. W. Kramer A. F. (2009). Age-related differences in the involvement of the prefrontal cortex in attentional control. Brain Cogn. 71, 328–335 10.1016/j.bandc.2009.07.005 19699019 PMC2783271

[B48] Prakash R. S. Heo S. Voss M. W. Patterson B. Kramer A. F. (2012). Age-related differences in cortical recruitment and suppression: implications for cognitive performance. Behav. Brain Res. 230, 192–200 10.1016/j.bbr.2012.01.058 22348896

[B49] Rajah M. N. D'Esposito M. (2005). Region-specific changes in prefrontal function with age: a review of PET and fMRI studies on working and episodic memory. Brain 128, 1964–1983 10.1093/brain/awh608 16049041

[B50] Reuter-Lorenz P. A. Cappell K. A. (2008). Neurocognitive aging and the compensation hypothesis. Curr. Dir. Psychol. Sci. 17, 177–182 10.1111/j.1467-8721.2008.00570.x

[B51] Reuter-Lorenz P. A. Park D. C. (2010). Human neuroscience and the aging mind: a new look at old problems. J. Gerontol. 65, 405–415 10.1093/geronb/gbq035 20478901 PMC2883872

[B52] Rottschy C. Langner R. Dogan I. Reetz K. Laird A. R. Schulz J. B. (2012). Modelling neural correlates of working memory: a coordinate-based meta-analysis. Neuroimage 60, 830–846 10.1016/j.neuroimage.2011.11.050 22178808 PMC3288533

[B53] Rypma B. D'Esposito M. (2000). Isolating the neural mechanisms of age-related changes in human working memory. Nat. Neurosci. 3, 509–515 10.1038/74889 10769393

[B54] Rypma B. Prabhakaran V. Desmond J. E. Gabrieli J. D. (2001). Age differences in prefrontal cortical activity in working memory. Psychol. Aging. 16, 371–384 10.1037/0882-7974.16.3.371 11554517

[B55] Schneider-Garces N. J. Gordon B. A. Brumback-Peltz C. R. Shin E. Lee Y. Sutton B. P. (2010). Span, CRUNCH, and beyond: working memory capacity and the aging brain. J. Cogn. Neurosci. 22, 655–669 10.1162/jocn.2009.21230 19320550 PMC3666347

[B56] Seidler R. D. Bernard J. A. Burutolu T. B. Fling B. W. Gordon M. T. Gwin J. T. (2010). Motor control and aging: links to age-related brain structural, functional, and biochemical effects. Neurosci. Biobehav. Rev. 34, 721–733 10.1016/j.neubiorev.2009.10.005 19850077 PMC2838968

[B57] Simon S. R. Meunier M. Piettre L. Berardi A. M. Segebarth C. M. Boussaoud D. (2002). Spatial attention and memory versus motor preparation: premotor cortex involvement as revealed by fMRI. J. Neurophysiol. 88, 2047–2057 10.1152/jn.00965.2001 12364527

[B58] Smith E. E. Jonides J. (1999). Storage and executive processes in the frontal lobes. Science 283, 1657–1661 10.1126/science.283.5408.1657 10073923

[B59] Stoodley C. J. (2012). The cerebellum and cognition: evidence from functional imaging studies. Cerebellum 11, 352–365 10.1007/s12311-011-0260-7 21373864

[B60] Talairach J. Tournoux P. (1988). Co-Planar Stereotactic Atlas of the Human Brain: 3-Dimensional Proportional System: An Approach to Cerebral Imaging. Stuttgart: Thieme

[B61] Tedesco A. M. Chiricozzi F. R. Clausi S. Lupo M. Molinari M. Leggio M. G. (2011). The cerebellar cognitive profile. Brain 134, 3672–3686 10.1093/brain/awr266 22036960

[B62] Toepper M. Gebhardt H. Beblo T. Thomas C. Driessen M. Bischoff M. (2010a). Functional correlates of distractor suppression during spatial working memory encoding. Neuroscience 165, 1244–1253 10.1016/j.neuroscience.2009.11.019 19925856

[B63] Toepper M. Markowitsch H. J. Gebhardt H. Beblo T. Thomas C. Gallhofer B. (2010b). Hippocampal involvement in working memory encoding of changing locations: an fMRI study. Brain Res. 1354, 91–99 10.1016/j.brainres.2010.07.065 20678490

[B64] Toepper M. Markowitsch H. Kater L. Gebhardt H. Beblo T. Bauer E. (2013). Neural correlates of impaired inhibitory processes in mild cognitive impairment. Behav. Neurol. 3, 27 10.3233/BEN-139900

[B65] Tzourio-Mazoyer N. Landeau B. Papathanassiou D. Crivello F. Etard O. Delcroix N. (2002). Automated anatomical labeling of activations in SPM using a macroscopic anatomical parcellation of the MNI MRI single-subject brain. Neuroimage 15, 273–289 10.1006/nimg.2001.0978 11771995

[B66] Zarahn E. Rakitin B. Abela D. Flynn J. Stern Y. (2007). Age-related changes in brain activation during a delayed item recognition task. Neurobiol. Aging 28, 784–798 10.1016/j.neurobiolaging.2006.03.002 16621168

