# Supplementary figures and images for: The impact of age on load-related dorsolateral prefrontal cortex activation
Source: Front Aging Neurosci. 2014 Feb 5;6:9. doi: 10.3389/fnagi.2014.00009 (PMC3913830; doi:10.3389/fnagi.2014.00009)

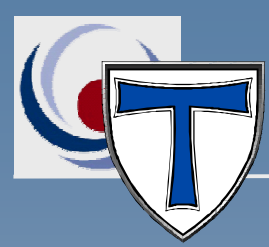

Corsi

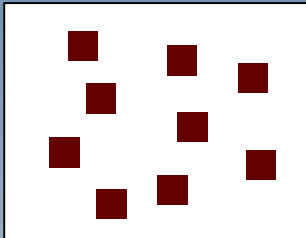

simplified

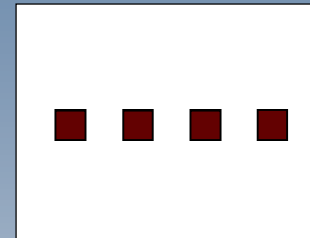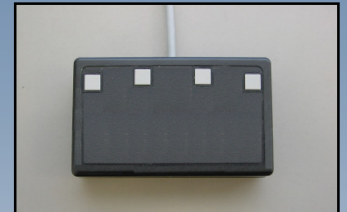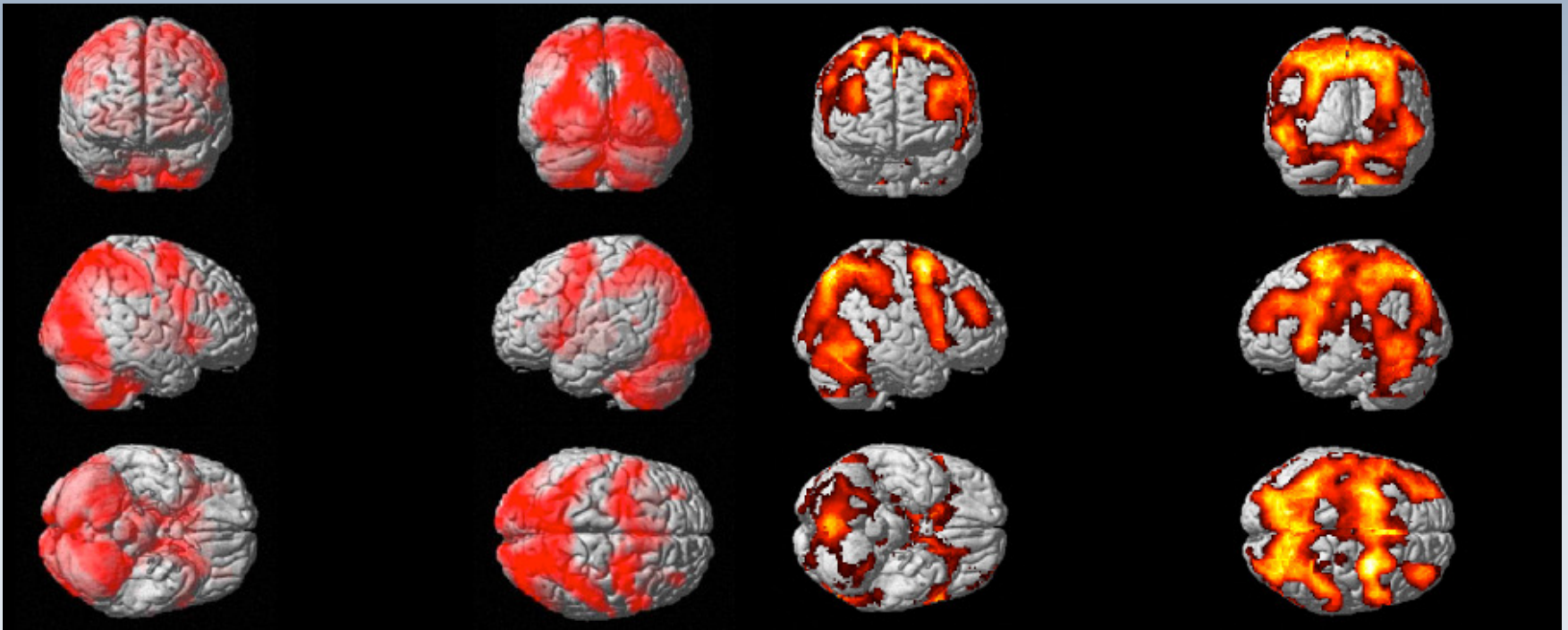

Supplement: Supplementary file 1 [file DataSheet1.ZIP › 67687_Toepper_Supplementary Material_1.PDF]

-38 36 28 (Nagel et al., PNAS 2009)

Peak voxel values x Age

---

Load 3

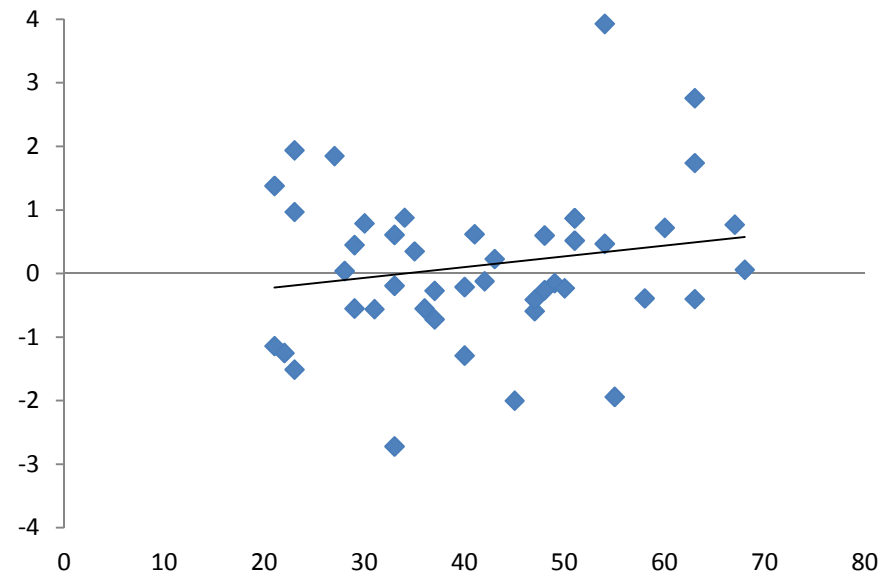

Load 4

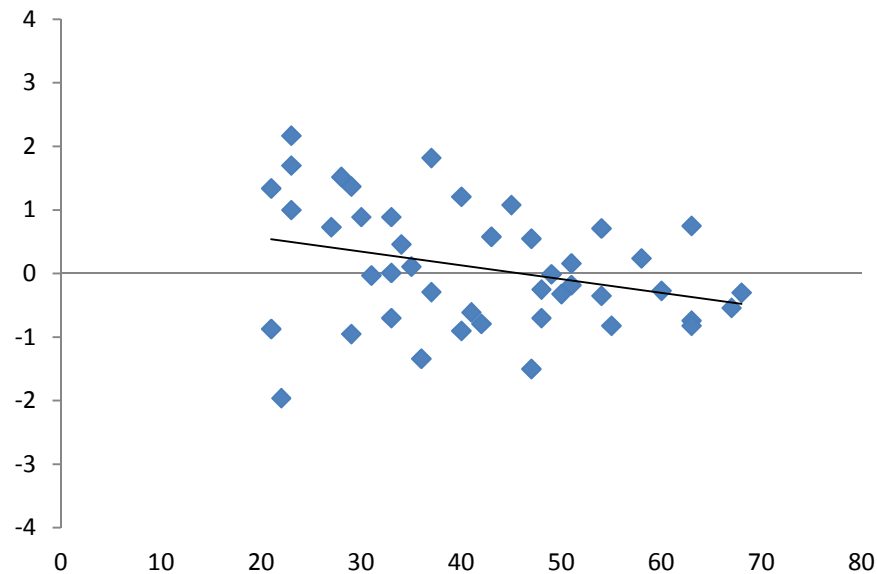

Load 5

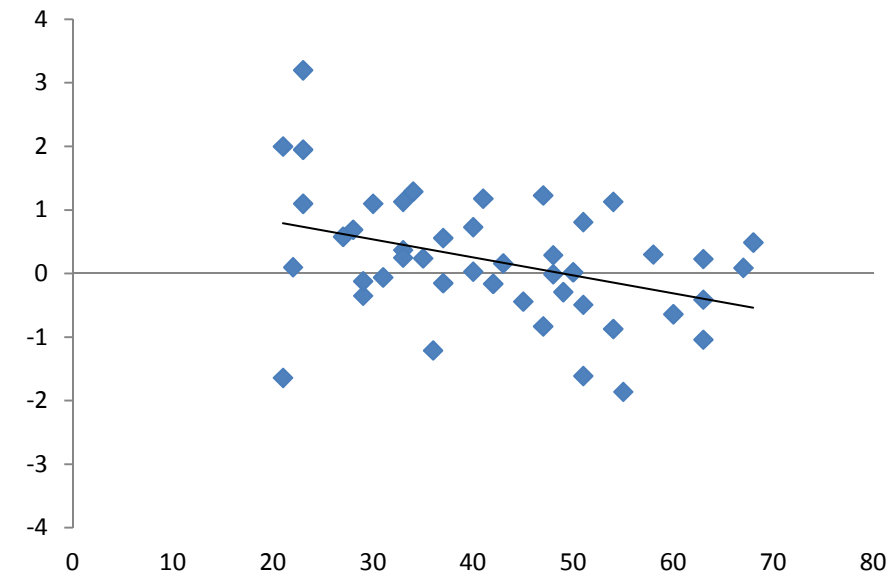

Supplement: Supplementary file 1 [file DataSheet1.ZIP › 67687_Toepper_Supplementary Material_2.PDF]
